# Rising DIP-weighted case mix, restructuring of cost composition, and expanding settlement deficits under diagnosis-intervention packet payment: evidence from case-level data in a tertiary grade a traditional Chinese medicine hospital in Anhui Province, China, 2022–2024

**DOI:** 10.3389/fpubh.2026.1868226

**Published:** 2026-09-08

**Authors:** Lina Zhu, Wenming Ban, Wei Liu, Haonan Shi

**Affiliations:** 1 The Seventh Affiliated Hospital of Anhui University of Chinese Medicine, Fuyang, Anhui, China; 2 School of Medicine, Shanghai University, Shanghai, China

**Keywords:** case mix, diagnosis-intervention packet (DIP), provider payment reform, settlement performance, tertiary traditional Chinese medicine hospital

## Abstract

**Background:**

Diagnosis-Intervention Packet (DIP) payment operates under regional global budgets in China. Multi-year evidence from tertiary traditional Chinese medicine (TCM) hospitals on settlement performance, cost composition, and departmental heterogeneity remains limited. We described temporal changes in DIP-weighted case mix, resource use, cost structure, and settlement performance and examined factors associated with case-level settlement deficit and any-deviation status.

**Methods:**

We retrospectively analysed inpatient DIP records from a tertiary Grade A TCM hospital in Anhui Province during 2022–2024. Outcomes were adjusted DIP settlement deficit (B_i_ < 0) and any-deviation status (high- or low-multiple versus normal). Logistic models used two-way cluster-robust standard errors for repeated admissions within patients and clustering within departments. Restricted cubic splines, year-specific upper-tail exclusions, and observed 30-day same-hospital readmission provided sensitivity and limited quality assessments.

**Results:**

Among 176,253 admissions from 118,520 patients, 27,912 patients (23.6%) had multiple admissions. From 2022 to 2024, median disease point value rose from 76.54 to 84.14 and case-mix index from 1.0777 to 1.2300, whereas mean length of stay fell from 9.47 to 8.71 days and mean cost per admission from 7,377 to 6,823 CNY. The aggregate examination and laboratory cost share rose from 19.30 to 22.65%; within-department changes accounted for +3.43 percentage points and departmental expenditure-mix changes for −0.08 percentage points. The unadjusted aggregate settlement shortfall increased from 16.40 million to 42.10 million CNY, and deficit cases increased from 56.0 to 63.2%. High-multiple classification declined from 10.4 to 2.6%, whereas low-multiple classification increased from 8.9 to 12.3%. Clustered models showed higher deficit odds in 2024 versus 2022 (OR 1.424, 95% CI 1.206–1.682) and with longer stay (linear-slope OR 1.123/day, 1.080–1.168), but lower odds with higher disease point value (OR 0.946/10 points, 0.930–0.962). Same-hospital 30-day readmission was 11.18, 10.89, and 11.54% across 2022–2024.

**Conclusion:**

At this single tertiary TCM hospital, higher DIP-weighted case mix and lower mean resource use coincided with less favourable administrative settlement performance. The observational design precludes causal attribution to DIP, and settlement deficit is not an accounting net loss. Findings support auditable department- and disease-group review and prospective evaluation incorporating TCM-service and clinical-quality indicators.

## Introduction

Reform of provider payment methods is a central component of China’s ongoing health system reform, with the dual aims of improving the efficiency of health insurance fund use and promoting more standardized clinical practice in healthcare institutions. In 2021, the National Healthcare Security Administration issued the Three-Year Action Plan for DRG/DIP Payment Reform (1), which explicitly required all pooling regions nationwide to implement DRG- or DIP-based payment reform by the end of 2024. The release of the Version 2.0 grouping scheme in 2024 further indicated that, by the end of 2023, more than 90% of pooling regions in China had already adopted DRG/DIP payment, and 26 provinces had achieved full coverage across all pooling regions within their jurisdictions (2). National implementation reports have described shorter average length of stay, lower time and cost consumption indices, and changes in hospital revenue composition following wider implementation of DRG/DIP payment (2). Because these reports are aggregate and do not isolate DIP from concurrent changes in patient mix, prices, referral patterns, or hospital management, they should be interpreted as implementation observations rather than as causal estimates.

Diagnosis-Intervention Packet (DIP) is a case-based payment model implemented under a regional global budget in China. In simplified form, the realized monetary value per point in pooling-region year t can be represented as V_t_ = B_t_/Σ_j_P_jt_, where B_t_ denotes the distributable DIP inpatient budget under local rules and Σ_j_P_jt_ denotes total recognized case points; case-level payment is then based on assigned disease points multiplied by V_t_, subject to locally applicable coefficients and settlement adjustments (3, 4). Thus, an institution may receive more assigned points without a commensurate improvement in its settlement balance when regional point volume, the budget, or local parameters also change. From a prospective-payment perspective, this changes the marginal return to additional services and may encourage resource conservation, while also creating possible incentives to alter coding, patient selection, treatment intensity, or service mix; these are competing mechanisms rather than presumed findings in the study hospital (5, 6). Anhui Province required all 17 pooling regions to initiate DRG/DIP reform by the end of 2022 and aimed for coverage of eligible institutions, diseases, and funds by the end of 2023 (7). The study hospital participated within the Fuyang pooling region during 2022–2024. Exact annual regional budget inputs, realized point values, institutional coefficients, and local deviation thresholds were not contained in the available extracts and were not imputed.

At the hospital level, reimbursement performance has been associated with CMI-standardized average cost per case, the proportion of deviation cases, institutional level, the proportion of class IV surgery, and insurance-case mix (8). This issue warrants particular attention in tertiary TCM hospitals, where inpatient pathways may combine biomedical diagnoses and procedures with syndrome differentiation, Chinese herbal medicine, acupuncture, tuina, and other TCM services. Existing Chinese evaluations in TCM-advantaged disease and TCM-hospital settings have reported heterogeneous changes in expenditure, length of stay, and the use of TCM-specific services under case-based payment (9, 10). Nevertheless, these findings do not establish that TCM services are systematically under-reimbursed, and any potential mismatch requires direct evaluation using TCM service-level costs and clinical outcomes.

Accordingly, this study addressed three questions: (1) how did DIP-weighted case mix, resource use, cost composition, and administrative settlement performance vary from 2022 to 2024; (2) was the increase in examination and laboratory cost share concentrated in particular departments or stable DIP groups, or was it primarily attributable to changes within units; and (3) which measured case characteristics were associated with a case-level DIP settlement deficit and any-deviation status? Given the descriptive and exploratory design, no confirmatory causal hypothesis was prespecified; the temporal and multivariable analyses were intended to characterize patterns and conditional associations. The study included only post-implementation observations from one hospital and no external comparison group and therefore was not designed to estimate the causal effect of DIP reform.

## Materials and methods

### Study design and data source

This was a single-center retrospective observational study conducted in a tertiary Grade A TCM hospital in Anhui Province, China. The study data were derived from inpatient case-level DIP records in the hospital information and health insurance settlement systems for settlement years 2022–2024. Eligible records had a non-missing settlement identifier and belonged to one of the three study years. Six non-case aggregate summary rows without settlement identifiers (two in each annual worksheet) were excluded; no patient-level case row was otherwise excluded, and no exclusions were applied by age, diagnosis, department, length of stay, cost, or deviation category. The final sample comprised 176,253 admissions. Admissions were linked across years using a hospital-held patient identifier that was converted to a pseudonymous integer cluster code before modeling; no direct identifiers were written to analytical outputs. All observations were post-implementation, with neither a pre-DIP baseline nor an external comparison hospital. The extracted variables included demographics, dates, discharge department, diagnosis/intervention information, settlement type, disease point value, deviation category, length of stay, expenditure and payment fields, the signed designated-provider adjustment, settlement balances, and component costs.

Disease point value was missing for 1,000 cases from 2022. These records were retained in analyses that did not require disease point value but were excluded from point-based indicators, the *post hoc* concordance assessment, and complete-case regression models containing disease point value. No missing values were imputed.

## Study variables

### Basic characteristics and case-mix indicators

Basic characteristics included sex, age, insurance category, and discharge department. Case-mix variables included disease-specific payment-pathway status, disease point value, deviation category, and the annual case mix index (CMI).

Administrative disease-specific payment-pathway status was harmonized from the corresponding annual field in the hospital settlement system (“single-disease” in 2022 and “whether single-disease” in 2023–2024). For analytic harmonization, 2022 records coded as “Yes” or “same disease, same coverage” were coded as affirmative and records coded as “No” as negative; the original Yes/No coding was retained in 2023–2024. This was an analytic recoding of administrative labels. It was not reconstructed from the number of diagnosis codes and should not be interpreted as distinguishing patients with a single diagnosis from those with multiple diagnoses, or as a measure of multimorbidity or clinical severity.

Settlement type was harmonized from the annual administrative field labelled “settlement type” in 2022–2023 and “disease type” in 2024. The harmonized categories were core disease, comprehensive disease, A + B disease, special-case negotiation, severe rehabilitation, and TCM-advantaged disease; core disease was the reference category in the multivariable models.

Disease point value was the case-level score assigned by the local DIP settlement system to the relevant diagnosis–intervention combination under the disease catalogue and adjustment rules applicable to the settlement year, consistent with the national DIP technical framework (3). A higher disease point value represented a greater relative payment weight and expected resource requirement within the DIP system. It was therefore treated as a payment-based case-mix measure rather than a direct measure of chart-reviewed clinical severity.

Deviation category was taken directly from the administrative field generated by the hospital’s DIP settlement system in each settlement year (“deviation flag” in 2022, “deviation” in 2023, and “cost category” in 2024), which classified cases as normal-, high-multiple-, or low-multiple cases. These terms reproduce the source-system labels and were not assumed to be exact synonyms for the national definitions of extremely low- or extremely high-cost cases.

The national rules were used only as a contextual policy benchmark. Under the National DIP Technical Specification Version 1.0 (3), let C_i_ denote total medical expenditure and Sg,t the payment standard for the assigned disease combination in settlement year t. An extremely low-cost case is defined by rᵢ = Cᵢ/Sg, t < 0.50 and an extremely high-cost case by rᵢ > 2.00; the complementary interval, 0.50 ≤ rᵢ ≤ 2.00, was treated as the national reference range. The same specification uses a different denominator when recalibrating abnormal-case weights, namely the previous-year mean total medical expenditure for the same disease among providers at the same institutional level. The screening cut-offs and the weight-recalibration formulas were therefore not conflated.

The subsequent national operating procedures (4) define deviation in relation to the previous-year mean expenditure for the same disease among same-level providers but do not prescribe a uniform national numerical cut-off; the calibration mechanism is operationalized by each pooling region. Because the exact year-specific local formula and supporting Fuyang policy documents were not contained in the analytical materials available for this study, the national 0.50 and 2.00 cut-offs were not assumed to be the official local thresholds for 2022–2024. The source-system categories were therefore retained without retrospective reassignment.

As a *post hoc* internal data-consistency check, we calculated q_i_ = C_i_/P_i_, where P_i_ is the assigned DIP point score and q_i_ is expressed in CNY per point. Inspection of the joint distribution of q_i_ and the pre-existing administrative labels showed transitions around 33.3 and 133.2 CNY/point; concordance was therefore summarized across the exploratory intervals q_i_ < 33.3, 33.3 ≤ q_i_ ≤ 133.2, and q_i_ > 133.2 CNY/point. These investigator-derived diagnostic cut-points were not obtained from, or validated against, an official local policy document and were not used to classify, reclassify, include, or exclude any case. Annual source fields, missingness, classification distributions, and concordance results are reported in Supplementary Table S1.

The annual hospital-level CMI was obtained directly from the hospital performance-management system. The national DIP technical specification identifies CMI as a case-mix metric (3). In this study, it was treated as a hospital-year, payment-weighted administrative indicator rather than a chart-validated measure of clinical severity. Because CMI was available only at the hospital-year level, it was used for descriptive trend reporting and was not included as a case-level covariate in the regression models.

### Resource utilization, cost structure, and settlement performance indicators

Indicators of resource utilization included length of stay and average hospitalization cost per case. We extracted total medical expenditure (Cᵢ), basic pooled-fund payment (Fᵢ), DIP settlement amount (Dᵢ), signed designated-provider adjustment (Aᵢ), system-recorded settlement balances, reimbursement ratio, western medicine cost, examination and laboratory cost, material cost, and therapeutic service income. The annual source files contained a single combined examination-and-laboratory cost field; examination and laboratory components could not be separated. The reimbursement ratio was taken from the annual settlement-system field; for 2022, it was calculated as the actual compensation amount divided by total medical expenditure. For admission i and component k, the case-level cost share was defined as Sᵢ_k_ = Cᵢ_k_/Cᵢ and was summarized by settlement year. Annual aggregate composition was calculated as S_k__t_ = Σᵢ∈_t_Cᵢ_k_/Σᵢ∈_t_Cᵢ. Other costs were calculated at the hospital-year level as the residual after subtracting the four reported components from total medical expenditure. Because a component share may change through its numerator, the total-expenditure denominator, or case mix, absolute component costs and proportional measures were interpreted jointly. The annual extracts did not contain consistently defined item-level fields for Chinese herbal medicine, TCM preparations, acupuncture, moxibustion, tuina, or other TCM-specific procedures. Therapeutic service income was therefore not treated as a proxy for TCM service income, and a distinct TCM-service cost share could not be calculated. Although “TCM-advantaged disease” was present as an administrative settlement-type category, it did not measure the actual intensity or proportion of TCM services.

For admission i, the pre-adjustment DIP settlement balance was calculated as G_i_ = D_i_−F_i_. The adjusted case-level DIP settlement balance was calculated as B_i_ = G_i_−A_i_ = D_i_−F_i_−A_i_, with A_i_ retained with its recorded sign. Blank adjustment fields, indicating that no adjustment was recorded, were treated as zero. A case-level DIP settlement deficit was defined as B_i_ < 0; otherwise, the case was classified as having no settlement deficit. This administrative settlement difference should not be interpreted as the hospital’s accounting net profit or loss because it does not incorporate the full operating-cost and revenue structure.

**Figure 1:**
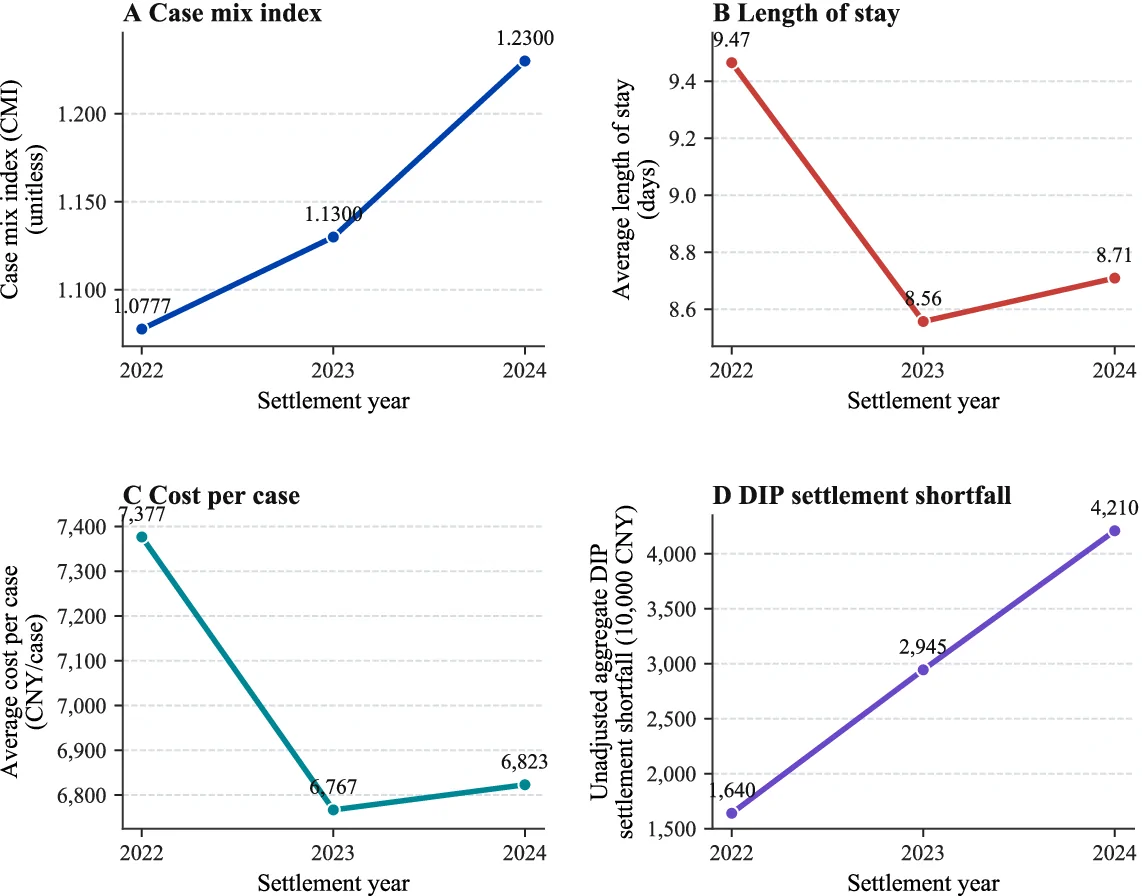
Trends in DIP-weighted case mix, resource utilization, and unadjusted aggregate settlement shortfall, 2022–2024 **(A)** CMI; **(B)** average length of stay (days); **(C)** average cost per case (CNY); and **(D)** unadjusted aggregate DIP settlement shortfall (10,000 CNY). CMI, case mix index; CNY, Chinese yuan; DIP, Diagnosis-Intervention Packet.

Derived indicators included cost per point (Cᵢ/Pᵢ, CNY/point), settlement balance per point (Bᵢ/Pᵢ, CNY/point), and the settlement-deficit rate (the proportion of cases with Bᵢ < 0). The annual unadjusted aggregate DIP settlement shortfall was calculated as Uᵧ = max[0, −ΣGᵢ] for all cases settled in year y; this hospital-year indicator corresponds to the unadjusted aggregate DIP settlement shortfall in Figure 1D and is distinct from the adjusted case-level outcome.

### Descriptive assessment of departmental heterogeneity

For descriptive assessment of departmental heterogeneity, we ranked departments by cumulative 2022–2024 admission volume after the prespecified name harmonization and retained the 20 highest-volume departments for the bubble chart. The x-axis represents mean disease point value, the y-axis mean adjusted DIP settlement balance (CNY per admission), bubble area cumulative case volume, and color the settlement-deficit rate. Direction-specific normal-, high-, and low-multiple distributions were also summarized for these departments. These comparisons were descriptive and were not interpreted as causal department effects.

### Decomposition and subgroup analysis of examination and laboratory cost composition

We summarized examination and laboratory costs by year as absolute case-level amounts, case-level shares, and aggregate expenditure-weighted shares. Department labels were harmonized using prespecified administrative renaming rules; departments with at least 100 admissions in every year were retained as stable units and all remaining units were pooled as Other. The 15 stable departments with the largest pooled admission volumes were displayed in Supplementary Figure S1. For department d and year t, rdt = ΣEdt/ΣCdt and wdt = ΣCdt/ΣCt, such that Rt = Σwdtrdt. The 2022–2024 change was decomposed exactly as:

$$\text{within}=\sum_{d}[\frac{w_{d,2022}+w_{d,2024}}{2}](r_{d,2024}-r_{d,2022})$$

and

$$\text{between}=\sum_{d}[\frac{r_{d,2022}+r_{d,2024}}{2}](w_{d,2024}-w_{d,2022})$$

We also performed an exact-code sensitivity analysis among DIP groups observed in all three years with at least 100 admissions per year and repeated the decomposition after excluding the year-specific upper 1% of total medical expenditure, length of stay, and both. These descriptive decompositions do not identify changes in service quantity, unit price, clinical necessity, or strategic provider behavior.

### Outcome definitions for multivariable analyses

Two prespecified binary outcomes were analyzed. The primary outcome was a case-level DIP settlement deficit, coded as 1 when B_i_ < 0 and as 0 when B_i_ ≥ 0. The secondary outcome was any-deviation status, coded as 1 for cases assigned by the settlement system to either the high-multiple or low-multiple group and as 0 for cases assigned to the normal group. The two deviation directions were pooled solely for an operational screening analysis intended to identify departure from the system-defined normal category. This pooling did not imply that high- and low-multiple cases shared the same clinical, coding, or economic mechanisms; the two categories were retained separately in descriptive analyses. Year, sex, age, insurance category, length of stay, disease point value, settlement type, administrative disease-specific payment-pathway status, and cost-structure indicators were included as covariates. Annual source fields, classification distributions, and *post hoc* concordance results are provided in Supplementary Table S1.

### Statistical analysis

All analyses were implemented in Python 3.12 using deterministic code. Categorical variables were summarized as *n* (%) and compared using χ^2^ tests; skewed continuous variables were summarized as median (P25, P75) and compared using Kruskal–Wallis H tests. Epsilon-squared was reported for the year difference in examination and laboratory cost share. Admissions were the analytical unit. Because 27,912 of 118,520 patients had multiple admissions and cases were nested within 51 harmonized departments, multivariable logistic regression used a two-way cluster-robust sandwich covariance estimator with patient and department clusters and the patient-by-department intersection correction. Adjusted odds ratios, 95% confidence intervals, and two-sided *p* values were reported. Department-level ICCs were estimated from intercept-only logistic random-intercept models by adaptive Gauss–Hermite quadrature and calculated as σ^2^/(σ^2^ + π^2^/3). Age and length of stay were represented linearly in the main tables for compact comparison. Nonlinearity was assessed using restricted cubic splines with knots at the 5th, 35th, 65th, and 95th percentiles; joint Wald tests of the nonlinear terms used the same two-way clustered covariance as the main models. Robustness was assessed after separately excluding the year-specific upper 1% of total cost, length of stay, and both. Disease point value was missing for 1,000 records from 2022, yielding 175,253 complete admissions for regression; no values were imputed. As a limited quality indicator, observed all-cause readmission to the same hospital 1–30 days after discharge was summarized by discharge year. For each index discharge, the first subsequent admission beginning strictly after discharge was identified; overlapping and same-day records were not counted as readmissions. Index discharges were restricted to January 1, 2022 through December 1, 2024 to provide a complete observation window through December 31, 2024. This indicator cannot capture readmissions to other hospitals or distinguish planned from unplanned readmissions. Statistical tests were two-sided with α = 0.05.

## Results

### General characteristics of the study population

From 2022 to 2024, the median age of hospitalized cases remained generally stable, whereas the median disease point value increased from 76.54 to 84.14, indicating an increase in case-level DIP payment weight. Over the same period, the proportion of cases managed under a disease-specific payment pathway decreased from 27.6 to 14.7%. This change represents a shift in an administrative payment-pathway classification and should not be interpreted as direct evidence of increasing multimorbidity or clinical severity. The proportion in the high-multiple group declined from 10.4 to 2.6%, whereas the low-multiple group increased from 8.9 to 12.7% and then decreased slightly to 12.3% in 2024 (Table 1).

**Table 1:** General characteristics and case-mix indicators of hospitalized cases, 2022–2024.

Variable	Category	2022 (*n* = 51,476)	2023 (*n* = 60,787)	2024 (*n* = 63,990)	χ^2^/H value	*p* value
Sex	Male	25,841 (50.2)	29,971 (49.3)	31,798 (49.7)	8.94^a^	0.011
	Female	25,635 (49.8)	30,816 (50.7)	32,192 (50.3)		
Insurance category	Employee insurance	3,302 (6.4)	3,939 (6.5)	4,075 (6.4)	0.65^a^	0.722
	Resident insurance	48,174 (93.6)	56,848 (93.5)	59,915 (93.6)		
Disease-specific payment-pathway status	Yes	14,224 (27.6)	11,121 (18.3)	9,421 (14.7)	3122.42^a^	<0.001
	No	37,252 (72.4)	49,666 (81.7)	54,569 (85.3)		
Deviation grouping	Normal group	41,527 (80.7)	50,114 (82.4)	54,462 (85.1)	3614.31^a^	<0.001
	High-multiple group	5,352 (10.4)	2,950 (4.9)	1,688 (2.6)		
	Low-multiple group	4,597 (8.9)	7,723 (12.7)	7,840 (12.3)		
Age, years		57.0 (40.0, 71.0)	57.0 (38.0, 71.0)	58.0 (41.0, 72.0)	108.76^b^	<0.001
Length of stay, days		8.0 (5.0, 11.0)	7.0 (5.0, 10.0)	7.0 (5.0, 11.0)	537.21^b^	<0.001
Disease point value		76.54 (53.76, 111.05)	77.14 (55.85, 117.46)	84.14 (60.77, 128.52)	1097.98^b^	<0.001

Values are *n* (%) or median (P25, P75). Disease point value was missing for 1,000 cases in 2022; its 2022 summary was based on 50,476 admissions. P25, 25th percentile; P75, 75th percentile.

a χ^2^ test.

b Kruskal–Wallis H test.

Among cases with calculable qᵢ, agreement between the exploratory intervals and the recorded labels was 94.3% (47,583/50,476) in 2022, 98.2% (59,674/60,787) in 2023, and 96.8% (61,932/63,990) in 2024. The ratio q_i_ could not be calculated for 1,000 otherwise eligible 2022 cases because disease point value was missing (Supplementary Table S1).

### Overall trends in DIP operation

Between 2022 and 2024, the annual hospital-level CMI increased from 1.0777 to 1.2300, representing a 14.13% increase in the DIP-weighted case mix. Because CMI is an administrative aggregate rather than a chart-validated severity measure, this trend should not be interpreted as direct evidence of increasing clinical severity (Figure 1A). Average length of stay decreased from 9.47 days to 8.56 days and then increased slightly to 8.71 days in 2024 (Figure 1B). Average cost per case declined from 7,377 CNY to 6,767 CNY and then increased slightly to 6,823 CNY (Figure 1C). The annual unadjusted aggregate DIP settlement shortfall increased from 16.40 million CNY to 42.10 million CNY, a 156.67% increase (Figure 1D).

### Annual changes in resource utilization, cost structure, and settlement performance

From 2022 to 2024, the median total medical expenditure and median DIP settlement amount both declined. However, the proportion of cases with a DIP settlement deficit increased from 56.0 to 63.2%, and the median adjusted DIP settlement balance became more negative, changing from −93.99 CNY to −218.38 CNY. Cost per point declined continuously, whereas settlement balance per point became progressively more negative, indicating that lower expenditure per point did not coincide with improved settlement performance. This temporal pattern describes a widening administrative payment difference but does not identify its cause. Median examination and laboratory cost was 1,214.78 CNY in 2022, 1,150.60 CNY in 2023, and 1,221.90 CNY in 2024 and therefore did not increase monotonically. In contrast, the median case-level share increased from 22.0 to 22.7 and 24.5%, respectively (Table 2). Between 2022 and 2024, the absolute median increased by only 7.13 CNY (0.59%), whereas median total medical expenditure decreased by 563.10 CNY (10.78%). Thus, the increase in share describes a relative change in cost composition and should not be interpreted as evidence of a monotonic increase in testing expenditure or utilization.

**Table 2:** Resource utilization, cost structure, and settlement performance of hospitalized cases, 2022–2024.

Variable	2022 (*n* = 51,476)	2023 (*n* = 60,787)	2024 (*n* = 63,990)	χ^2^/H value	*p* value
DIP settlement deficit, yes	28,814 (56.0)	36,664 (60.3)	40,451 (63.2)	625.19^a^	<0.001
DIP settlement deficit, no	22,662 (44.0)	24,123 (39.7)	23,539 (36.8)		
Total medical expenditure, CNY	5221.94 (3484.03, 7979.58)	4738.59 (3125.29, 7278.38)	4658.85 (3126.80, 7202.48)	833.24^b^	<0.001
DIP settlement amount, CNY	2862.20 (1906.85, 4481.13)	2491.32 (1657.48, 3973.57)	2399.44 (1567.78, 3777.02)	1940.23^b^	<0.001
Adjusted DIP settlement balance, CNY	−93.99 (−1053.56, 546.03)	−144.14 (−900.91, 381.49)	−218.38 (−968.42, 256.88)	549.75^b^	<0.001
Reimbursement ratio (0–1)	0.600 (0.539, 0.606)	0.582 (0.516, 0.648)	0.586 (0.525, 0.648)	151.48^b^	<0.001
Cost per point value, CNY/point	66.17 (48.72, 90.84)	56.95 (42.81, 76.30)	51.95 (40.32, 69.64)	6376.12^b^	<0.001
Settlement balance per point, CNY/point	−1.06 (−15.25, 6.75)	−1.74 (−11.01, 4.81)	−2.69 (−10.51, 3.08)	348.74^b^	<0.001
Western medicine cost, CNY	453.63 (176.22, 1042.14)	385.15 (158.86, 914.07)	392.37 (157.44, 913.58)	338.36^b^	<0.001
Examination and laboratory cost, CNY	1214.78 (730.77, 1883.79)	1150.60 (649.40, 1900.70)	1221.90 (670.10, 1973.65)	142.40^b^	<0.001
Material cost, CNY	51.00 (4.13, 388.50)	64.54 (7.65, 324.16)	49.07 (6.75, 242.78)	115.32^b^	<0.001
Therapeutic service income, CNY	2236.85 (1300.20, 3859.65)	2045.60 (1203.30, 3594.75)	1958.30 (1165.95, 3501.78)	450.88^b^	<0.001
Western medicine cost share (proportion, 0–1)	0.085 (0.042, 0.161)	0.082 (0.042, 0.154)	0.083 (0.041, 0.152)	41.61^b^	<0.001
Examination and laboratory cost share (proportion, 0–1)	0.220 (0.140, 0.343)	0.227 (0.144, 0.357)	0.245 (0.153, 0.388)	676.76^b^	<0.001
Material cost share (proportion, 0–1)	0.011 (0.001, 0.069)	0.016 (0.001, 0.064)	0.011 (0.002, 0.052)	147.87^b^	<0.001
Therapeutic service income share (proportion, 0–1)	0.450 (0.321, 0.570)	0.453 (0.330, 0.575)	0.436 (0.315, 0.570)	116.66^b^	<0.001

Values are *n* (%) or median (P25, P75). A DIP settlement deficit was defined as an adjusted DIP settlement balance <0 CNY. The reimbursement ratio and component cost shares are reported on a 0–1 scale. CNY, Chinese yuan; DIP, Diagnosis-Intervention Packet; P25, 25th percentile; P75, 75th percentile.

a χ^2^ test.

b Kruskal–Wallis H test.

**Figure 2:**
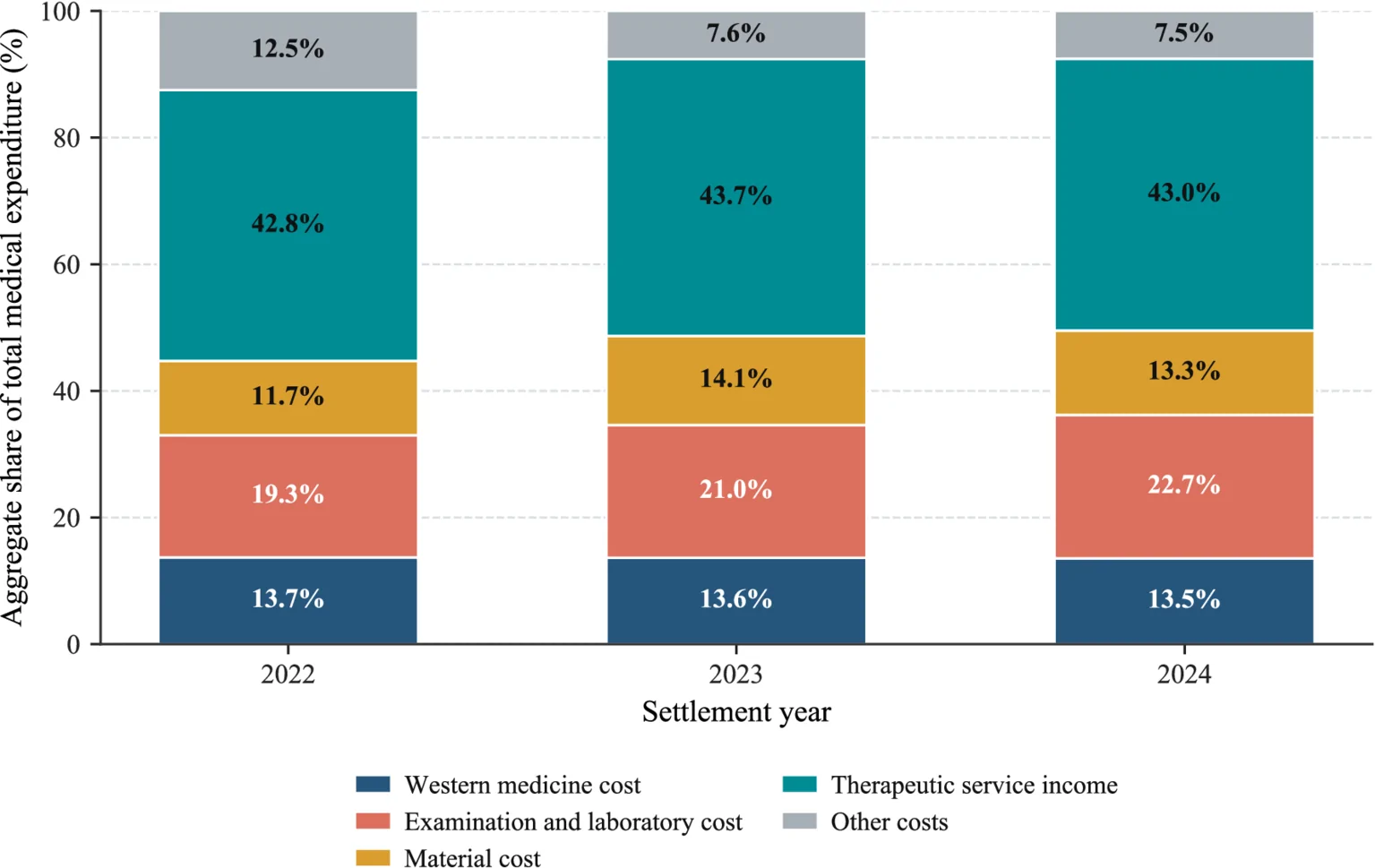
Aggregate inpatient cost composition as a percentage of total medical expenditure, 2022–2024. Other costs represent the residual after subtracting Western medicine, examination and laboratory, material, and therapeutic service costs from total medical expenditure.

At the aggregate hospital level, the examination and laboratory share increased from 19.30% in 2022 to 22.65% in 2024 (+3.36 percentage points), the largest increase among the four reported components. Over the same period, the residual other-cost share decreased from 12.50 to 7.53% (−4.97 percentage points). These aggregate expenditure-weighted shares differ from the medians of case-level shares in Table 2. Across individual admissions, the year difference in the examination and laboratory share was statistically significant (H = 676.76, *p* < 0.001), but the rank-based effect size was small (ε^2^ = 0.0038). Exact accounting decomposed the 2022–2024 increase into +1.73 percentage points corresponding to the change in mean examination and laboratory cost and +1.63 percentage points corresponding to the change in mean total expenditure (Figure 2; Supplementary Table S2; Supplementary Figure S2A).

### Department- and disease-group patterns in examination and laboratory cost composition

The examination and laboratory cost share increased at both the case and hospital levels, but the absolute amount per admission did not increase monotonically. Median examination and laboratory cost was 1,214.78 CNY in 2022, 1,150.60 CNY in 2023, and 1,221.90 CNY in 2024, whereas the median case-level share increased from 22.0 to 22.7 and 24.5%, respectively. At the aggregate hospital level, the share increased from 19.30 to 22.65% (+3.36 percentage points). An exact numerator-denominator accounting decomposed the increase into +1.73 percentage points corresponding to the change in mean examination and laboratory cost and +1.63 percentage points corresponding to the change in mean total expenditure (Supplementary Table S2; Supplementary Figure S2A).

After harmonization, 41 stable departments with at least 100 admissions in each year covered 98.5, 99.9, and 96.8% of admissions in 2022, 2023, and 2024, respectively. The aggregate examination and laboratory share increased in 34 departments and decreased in seven (Supplementary Table S3; Supplementary Figure S1). The hospital-level increase of 3.36 percentage points was decomposed into a + 3.43-percentage-point within-department component and a − 0.08-percentage-point between-department expenditure-mix component (Supplementary Figure S2B). Thus, changes in departmental expenditure mix slightly offset rather than explained the hospital-wide increase. In the exact-code sensitivity analysis, 39 of 48 high-volume DIP groups showed an increase, although these groups covered only 35.6% of admissions in 2024 (Supplementary Table S4). Excluding the year-specific upper 1% of total cost, length of stay, or both produced aggregate increases of 3.31, 3.23, and 3.26 percentage points, respectively (Supplementary Table S5). The prespecified department-name harmonization rules are provided in Supplementary Table S6.

These findings indicate that the accounting increase in the aggregate examination and laboratory cost share was dominated by the within-department component. They do not establish changes in test volume, unit prices, clinical necessity, or deliberate cost shifting.

### Heterogeneity in DIP operational performance across major departments

Marked heterogeneity in DIP operational performance was observed across the 10 highest-volume departments. Cardiology Department I and Pediatrics Department II had median adjusted DIP settlement balances of 98.65 CNY and 73.03 CNY per admission, respectively, and settlement-deficit rates of 46.0 and 44.1%. In contrast, Anorectal Surgery had a median adjusted balance of −566.09 CNY per admission and a settlement-deficit rate of 74.3%; Oncology had a settlement-deficit rate of 71.2%. Surgery Department I and Cardiology Department I had relatively high median disease point values of 102.05 and 95.93, respectively, whereas the two pediatric departments had lower disease point values and lower expenditure. Thus, departments differed in DIP-assigned payment weights, resource input, and settlement performance (Table 3).

**Table 3:** DIP operational performance in the 10 highest-volume departments, 2022–2024.

Department	Cases, *n* (%)	Disease point value	Total medical expenditure, CNY	Adjusted DIP settlement balance, CNY	Cases with a DIP settlement deficit, *n* (%)
Pediatrics department I	10,143 (5.8)	53.76 (40.89, 66.18)	2,476.34 (1,908.90, 3,168.92)	−6.21 (−229.94, 317.41)	5,150 (50.8)
Oncology department	9,959 (5.7)	83.23 (37.28, 131.72)	4,793.75 (3,316.97, 7,353.03)	−293.13 (−896.26, 119.95)	7,086 (71.2)
Neurology department I	8,718 (4.9)	59.98 (55.17, 109.90)	4,314.75 (3,190.96, 6,482.71)	−248.67 (−1,060.24, 249.74)	5,499 (63.1)
Neurology department II	8,655 (4.9)	65.13 (55.17, 109.90)	4,228.61 (3,198.13, 6,394.65)	−137.40 (−850.63, 380.52)	4,949 (57.2)
Cardiology department I	7,713 (4.4)	95.93 (66.18, 129.85)	5,152.46 (3,727.03, 8,057.50)	98.65 (−531.95, 863.56)	3,549 (46.0)
Otolaryngology department	7,695 (4.4)	88.07 (58.58, 110.02)	5,311.94 (3,265.39, 6,744.67)	−54.52 (−699.85, 417.08)	4,317 (56.1)
Surgery department I	7,691 (4.4)	102.05 (68.51, 156.16)	6,087.82 (4,437.52, 8,191.51)	−183.05 (−1,013.80, 381.30)	4,705 (61.2)
Pulmonology department	7,307 (4.1)	77.14 (75.64, 91.06)	5,292.26 (3,862.43, 7,280.32)	−67.69 (−993.57, 665.35)	3,830 (52.4)
Anorectal surgery department	6,054 (3.4)	59.06 (35.85, 73.11)	4,710.01 (3,010.68, 5,611.68)	−566.09 (−1,299.12, 14.83)	4,501 (74.3)
Pediatrics department II	5,905 (3.4)	53.76 (38.21, 66.18)	2,432.14 (1,889.09, 3,088.82)	73.03 (−192.26, 518.72)	2,606 (44.1)

Departments were ranked by cumulative admission volume in 2022–2024 after applying the prespecified department-name harmonization. Values are *n* (%) or median (P25, P75). CNY, Chinese yuan; DIP, Diagnosis-Intervention Packet; P25, 25th percentile; P75, 75th percentile.

**Figure 3:**
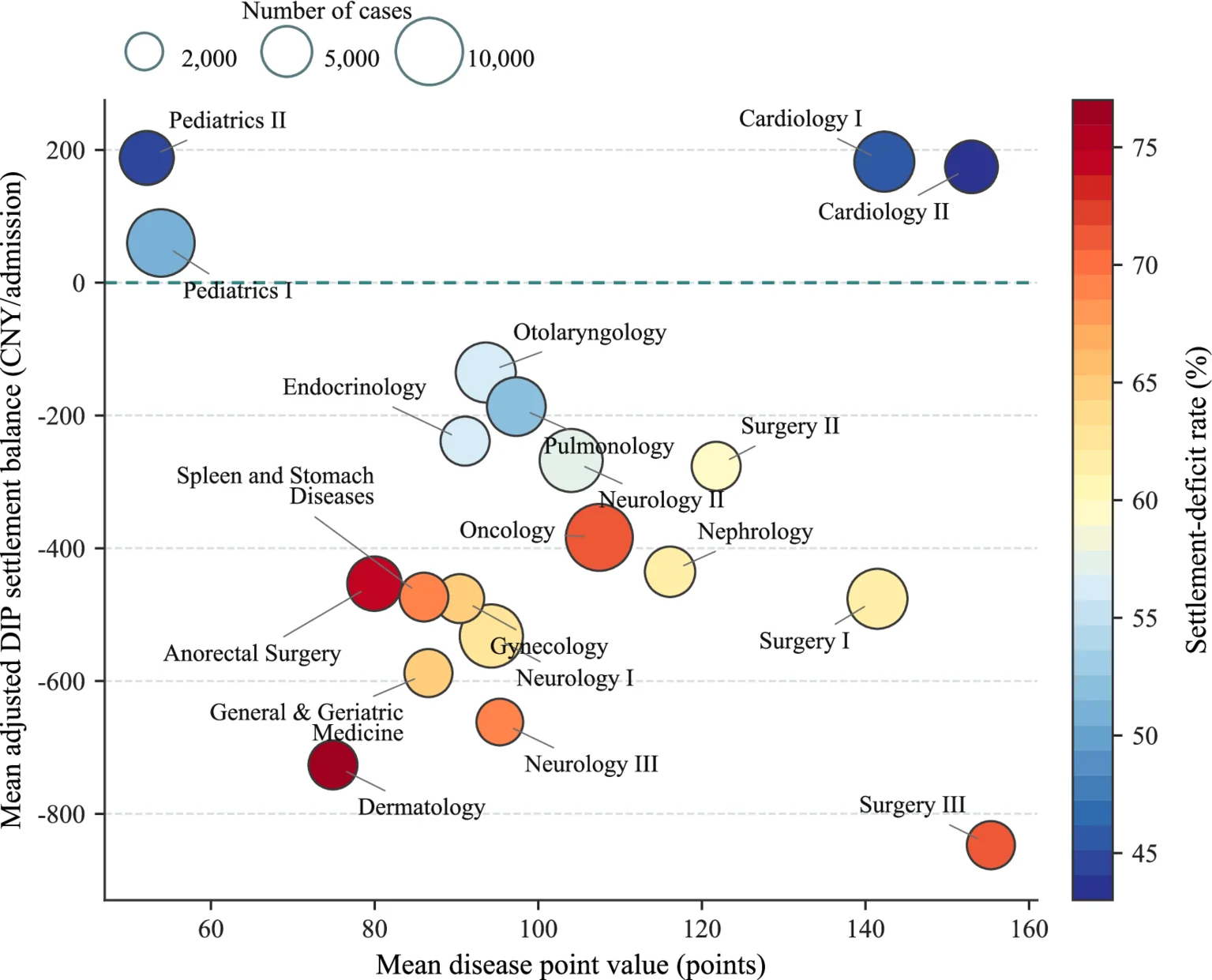
Mean disease point value and adjusted DIP settlement balance across the 20 highest-volume departments, 2022–2024. Bubble area represents cumulative admission volume, color represents the settlement-deficit rate, and the dashed horizontal line marks zero adjusted balance. CNY, Chinese yuan; DIP, Diagnosis-Intervention Packet.

A bubble chart was constructed for the 20 highest-volume departments after applying the same prespecified harmonization used in all department analyses. Cardiology Departments I and II had mean disease point values of 142.30 and 152.96 and mean adjusted balances of 182.25 and 174.47 CNY per admission, respectively. Surgery Department III, Dermatology, Neurology Department III, and General & Geriatric Medicine had mean adjusted balances of −847.11, −726.33, −661.63, and −587.93 CNY per admission, respectively. These mean profiles do not measure each department’s contribution to the hospital-wide aggregate shortfall. The highest settlement-deficit rates were observed in Dermatology (76.2%), Anorectal Surgery (74.3%), Oncology (71.2%), and Surgery Department III (70.7%) (Figure 3). Direction-specific deviation patterns also differed: the high-multiple proportion was 22.3% in Anorectal Surgery, whereas the low-multiple proportion was 26.5% in Nephrology and 27.1% in Surgery Department III. These descriptive patterns identify targets for coding and clinical review but do not establish either gaming or efficiency gains (Supplementary Table S7).

### Multivariable analysis of factors associated with a case-level DIP settlement deficit

The 176,253 admissions represented 118,520 patients; 27,912 patients (23.6%) had multiple admissions, accounting for 85,645 admissions (48.6%), and 17,120 patients were observed in more than one settlement year. In intercept-only department logistic random-intercept models estimated by adaptive Gauss–Hermite quadrature, the latent-scale ICC was 0.080 for settlement deficit and 0.107 for any-deviation status, supporting adjustment for within-department correlation (Supplementary Table S8).

After covariate adjustment and two-way patient- and department-clustered inference, the odds of a DIP settlement deficit were higher in 2023 (OR = 1.206, 95% CI: 1.033–1.408) and 2024 (OR = 1.424, 95% CI: 1.206–1.682) than in 2022. In the linear-slope specification, each additional hospital day was associated with higher odds (OR = 1.123, 95% CI: 1.080–1.168), whereas each 10-point increase in disease point value was associated with lower odds (OR = 0.946, 95% CI: 0.930–0.962). Cases managed under a disease-specific payment pathway also had lower odds (OR = 0.366, 95% CI: 0.276–0.485). Western medicine and material cost shares were not statistically distinguishable from the null after clustered inference. Examination/laboratory share (OR = 0.879 per 10 percentage points, 95% CI: 0.785–0.984) and therapeutic service income share (OR = 0.860, 95% CI: 0.793–0.933) were inversely associated, but these compositional coefficients should not be interpreted causally (Table 4).

**Table 4:** Multivariable logistic regression of factors associated with a case-level DIP settlement deficit, with patient- and department-clustered standard errors.

Variable	Reference	Compared level	β	SE	Wald χ^2^	OR	95% CI	P value
Year	2022	2023	0.187	0.079	5.63	1.206	1.033–1.408	0.018
	2022	2024	0.354	0.085	17.35	1.424	1.206–1.682	<0.001
Sex	Male	Female	0.004	0.035	0.01	1.004	0.938–1.075	0.904
Insurance category	Resident insurance	Employee insurance	−0.584	0.063	85.53	0.558	0.493–0.631	<0.001
Disease-specific payment-pathway status	No	Yes	−1.006	0.144	48.86	0.366	0.276–0.485	<0.001
Age (per 10-year increase)	—	—	0.018	0.024	0.58	1.018	0.972–1.066	0.445
Length of stay (per 1-day increase)	—	—	0.116	0.020	33.89	1.123	1.080–1.168	<0.001
Disease point value (per 10-point increase)	—	—	−0.055	0.009	40.29	0.946	0.930–0.962	<0.001
Western medicine cost share (per 10-percentage-point increase)	—	—	0.034	0.058	0.34	1.035	0.923–1.160	0.559
Examination and laboratory cost share (per 10-percentage-point increase)	—	—	−0.129	0.057	5.02	0.879	0.785–0.984	0.025
Material cost share (per 10-percentage-point increase)	—	—	0.068	0.078	0.77	1.071	0.920–1.247	0.379
Therapeutic service income share (per 10-percentage-point increase)	—	—	−0.150	0.041	13.14	0.860	0.793–0.933	<0.001

Complete-case analysis included 175,253 admissions (2022, *n* = 50,476; 2023, *n* = 60,787; 2024, *n* = 63,990); no missing values were imputed. The model was additionally adjusted for settlement type (core disease [reference], comprehensive disease, A + B disease, special-case negotiation, severe rehabilitation, and TCM-advantaged disease); these coefficients are not shown. Standard errors, confidence intervals, Wald χ^2^ statistics, and *p* values use two-way patient- and department-clustered inference. Age and length of stay are linear-slope summaries; clustered spline analyses are reported in Supplementary Table S9. CI, confidence interval; DIP, Diagnosis-Intervention Packet; OR, odds ratio; SE, standard error; TCM, traditional Chinese medicine.

### Multivariable analysis of factors associated with any-deviation status

With two-way patient- and department-clustered inference, the odds of any-deviation status were lower in 2023 (OR = 0.855, 95% CI: 0.752–0.972) and 2024 (OR = 0.659, 95% CI: 0.540–0.803) than in 2022. Cases managed under a disease-specific payment pathway had lower odds (OR = 0.384, 95% CI: 0.199–0.739). Western medicine share was positively associated (OR = 1.188 per 10 percentage points, 95% CI: 1.030–1.370) and material share inversely associated (OR = 0.744, 95% CI: 0.624–0.888). Examination/laboratory and therapeutic service income shares were not statistically distinguishable from the null. The OR for disease point value was 1.014 per 10-point increase (95% CI: 1.005–1.022). Although statistically detectable, this estimate was very close to the null and should not be interpreted as economically or operationally important in isolation (Table 5).

**Table 5:** Multivariable logistic regression of factors associated with any-deviation status, with patient- and department-clustered standard errors.

Variable	Reference	Compared level	β	SE	Wald χ^2^	OR	95% CI	*p* value
Year	2022	2023	−0.157	0.065	5.73	0.855	0.752–0.972	0.017
	2022	2024	−0.418	0.101	17.06	0.659	0.540–0.803	<0.001
Sex	Male	Female	−0.131	0.058	5.12	0.877	0.783–0.983	0.024
Insurance category	Resident insurance	Employee insurance	−0.083	0.097	0.73	0.920	0.761–1.114	0.394
Disease-specific payment-pathway status	No	Yes	−0.958	0.334	8.21	0.384	0.199–0.739	0.004
Age (per 10-year increase)	—	—	−0.024	0.018	1.69	0.977	0.942–1.012	0.194
Length of stay (per 1-day increase)	—	—	−0.014	0.014	0.91	0.986	0.959–1.015	0.340
Disease point value (per 10-point increase)	—	—	0.013	0.004	9.92	1.014	1.005–1.022	0.002
Western medicine cost share (per 10-percentage-point increase)	—	—	0.172	0.073	5.63	1.188	1.030–1.370	0.018
Examination and laboratory cost share (per 10-percentage-point increase)	—	—	0.126	0.069	3.30	1.134	0.990–1.299	0.069
Material cost share (per 10-percentage-point increase)	—	—	−0.295	0.090	10.70	0.744	0.624–0.888	0.001
Therapeutic service income share (per 10-percentage-point increase)	—	—	0.042	0.047	0.79	1.043	0.950–1.144	0.375

Complete-case analysis included 175,253 admissions (2022, *n* = 50,476; 2023, *n* = 60,787; 2024, *n* = 63,990); no missing values were imputed. The model was additionally adjusted for settlement type; these coefficients are not shown. Standard errors, confidence intervals, Wald χ^2^ statistics, and *p* values use two-way patient- and department-clustered inference. Age and length of stay are linear-slope summaries; spline analyses are reported in Supplementary Table S9. CI, confidence interval; DIP, Diagnosis-Intervention Packet; OR, odds ratio; SE, standard error; TCM, traditional Chinese medicine.

Using the same two-way patient- and department-clustered covariance as the main models, restricted cubic spline analyses provided evidence of nonlinearity for length of stay in both the settlement-deficit model (Wald χ^2^ = 18.68, *p* < 0.001) and the any-deviation model (Wald χ^2^ = 68.86, *p* < 0.001), but not for age (*p* = 0.065 and *p* = 0.467, respectively). Thus, the linear length-of-stay ORs in Tables 4, 5 are compact average-slope summaries and should not be assumed constant over the observed range. After excluding the year-specific upper 1% of total cost, length of stay, or both, the directions of the year, disease-specific payment-pathway, and disease-point associations were unchanged. Some cost-share associations crossed the null, reinforcing their exploratory interpretation (Supplementary Table S10).

### Limited quality assessment

Observed 30-day all-cause readmission to the same hospital was 11.18% (5,756/51,474) for discharge year 2022, 10.89% (6,622/60,785) for 2023, and 11.54% (6,809/59,026 eligible index discharges) for 2024. The year difference was statistically detectable (χ^2^ = 12.42, *p* = 0.002), but the maximum absolute difference was only 0.64 percentage points. This measure did not capture admissions to other hospitals or distinguish planned from unplanned readmissions (Supplementary Table S11).

## Discussion

Using case-level records from a single tertiary Grade A TCM hospital, we observed that increases in disease point value and CMI from 2022 to 2024 coincided with shorter average length of stay and lower mean inpatient cost, but also with a larger unadjusted aggregate settlement shortfall, a higher proportion of settlement-deficit cases, and a more negative median adjusted settlement balance. These measures should not be collapsed into a single construct: lower resource use does not demonstrate improved efficiency, particularly because the only available quality measure was observed 30-day same-hospital readmission and it could not capture other hospitals or distinguish planned from unplanned events. The adjusted settlement balance is an administrative payment difference rather than accounting net profit or loss. Quasi-experimental studies have reported reductions in expenditure or length of stay in some DIP settings (5, 11), whereas systematic-review evidence relating prospective payment to clinical quality remains inconclusive (12). Because this study included neither a pre-DIP period nor a contemporaneous comparison hospital, the temporal patterns cannot be attributed causally to DIP. Concurrent changes in patient mix, referral patterns, service prices, local catalogues, institutional coefficients, the regional budget, or realized point value remain plausible explanations.

The increase in median disease point value and CMI indicates an upward shift in payment-weighted case mix rather than chart-validated clinical complexity. Evidence from other DIP settings suggests that changes in case weight, expenditure, and treatment intensity vary across insurance groups and hospital levels (5, 6, 11). In the present data, the high-multiple proportion decreased while the low-multiple proportion increased, and their departmental distributions differed. Patient linkage allowed repeated admissions to be clustered and a limited same-hospital readmission measure to be calculated, but the data lacked chart review, coding audits, verified year-specific local rules, and planned-versus-unplanned readmission status. Genuine efficiency gains, coding changes, patient selection, split admissions, and payment-group mismatch therefore remain competing explanations rather than study findings. The low-multiple label is not direct evidence of either improved efficiency or policy gaming, and any-deviation status remains an operational screening indicator rather than a validated performance measure.

Three findings constrain the interpretation of the increase in the examination and laboratory cost share. First, the absolute median examination and laboratory cost did not increase monotonically, whereas both the case-level and aggregate cost shares increased. Second, the exact accounting decomposition attributed +1.73 percentage points to the component-cost term and +1.63 percentage points to the total-expenditure denominator term. Third, the share increased in 34 of the 41 stable departments. The within-department component accounted for +3.43 percentage points of the hospital-level change, whereas the between-department expenditure-mix component was slightly negative at −0.08 percentage points. A recent study from another tertiary hospital in Anhui Province likewise found that changes in inpatient cost composition after DIP implementation differed between surgical and non-surgical cases (13). However, external evidence on provider responses is mixed. One DRG evaluation reported increased diagnostic-test expenditure and behavioral signals consistent with cost shifting, patient selection, and upcoding (14), whereas a city-wide DIP study found no increase in diagnostic weight in secondary or tertiary hospitals and attributed the increase in total weight primarily to treatment intensity (6). In addition, a national study of public-hospital price reform showed that changes in regulated service prices can alter expenditure composition and that expenditure changes alone cannot establish corresponding changes in service volume (15). A component share may also change through either its numerator or its total-expenditure denominator. The present findings therefore indicate a broad accounting change dominated by within-department variation, but they do not identify increased testing volume, greater clinical need, tariff changes, technology substitution, or strategic cost shifting as its cause.

Departmental results showed substantial descriptive heterogeneity in both settlement profiles and deviation direction. Cardiology Departments I and II had relatively favorable mean adjusted balances despite high disease point values, whereas Surgery Department III, Dermatology, Neurology Department III, and General & Geriatric Medicine had less favorable mean profiles. High-multiple classification was relatively prominent in Anorectal Surgery, whereas low-multiple classification was relatively prominent in Nephrology and Surgery Department III. These comparisons can prioritize chart, coding, and pathway review, but mean balances do not measure contribution to the aggregate shortfall, and the direction-specific labels do not reveal mechanism. Department-specific pathways, item-level use, examination indications, prices, and quality outcomes beyond the limited hospital-wide readmission indicator were unavailable.

The TCM setting is clinically and operationally relevant, but it is not, by itself, an explanation for the observed settlement pattern. TCM hospitals may deliver mixed inpatient pathways combining conventional diagnoses and procedures with syndrome differentiation, Chinese herbal medicine, acupuncture, moxibustion, tuina, and other labor-intensive nonpharmacological techniques. Recent Chinese evaluations have reported setting-specific changes in expenditure, length of stay, and selected TCM-service indicators following case-based payment reform (9, 10). These findings indicate that payment design may interact with TCM clinical pathways, but they cannot be assumed to apply to the present hospital. The available extracts did not separately identify Chinese herbal medicine, TCM preparations, acupuncture, moxibustion, tuina, or other TCM-specific service costs, and therapeutic service income cannot be treated as a surrogate for TCM service intensity. The study also lacked a general-hospital comparator. Consequently, the results do not demonstrate that the observed settlement deficits were caused by under-valuation of TCM services or that the temporal pattern was unique to TCM hospitals.

In the linear-slope multivariable model, longer length of stay was associated with higher odds of a case-level settlement deficit, whereas higher disease point value and administrative disease-specific payment-pathway status were associated with lower odds. Two-way clustered inference materially widened several confidence intervals and removed statistical significance for some cost-share coefficients, illustrating why the large number of admissions should not be confused with an equally large number of independent observations. Clustered spline analyses showed nonlinearity for length of stay, but not age, in both outcomes. Consequently, the linear length-of-stay ORs are compact average-slope summaries rather than constant marginal effects, and the models do not establish that shortening a stay, changing assigned points, or changing an administrative pathway would improve settlement performance. The any-deviation OR of 1.014 per 10-point increase in disease point value was statistically detectable but very close to the null. Cost-share coefficients are conditional and compositional and may reflect unmeasured case mix, clinical need, price differences, or denominator changes.

The findings support staged and auditable responses at the study hospital rather than broad recommendations for all tertiary hospitals in Anhui Province. First, department- and DIP-group monitoring should report disease point value, length of stay, adjusted balance, case volume, and high- and low-multiple directions separately; aggregate negative balances should be reported alongside means. Second, prolonged stays should undergo structured clinical review before pathway changes are piloted. The observed same-hospital 30-day readmission rate varied only modestly across years, but future evaluations should distinguish planned from unplanned readmission and add mortality, complications, and patient-safety indicators (12, 16). Third, the hospital should prospectively record the numbers of cases eligible for, submitted to, approved through, and additionally reimbursed under special-case negotiation, with final balances. DIP Version 2.0 describes special-case negotiation for prolonged-stay, high-cost, new-technology, complex, critical, or multidisciplinary cases (2). Although special-case negotiation appeared as an administrative settlement-type category, the available extracts did not document eligibility assessment, submission, formal approval, incremental reimbursement, or final negotiated outcomes; therefore, the extent of uptake and any resulting benefit could not be evaluated. Fourth, TCM-specific monitoring should separate administrative TCM-advantaged disease status from actual item-level TCM service use and link both to cost and clinical outcomes. Anhui’s July 2024 pilot for six TCM-advantaged fracture conditions is a setting for prospective evaluation (17), not an explanation for the full 2022–2024 trend.

### Strengths and limitations

An important strength is the use of a large case-level dataset with explicit settlement definitions, department-level analyses, exact cost-share decomposition, patient linkage, cluster-robust inference, nonlinear checks, upper-tail sensitivity analyses, and a limited quality indicator. Several limitations remain. First, this single tertiary TCM hospital represents one pooling region; catalogues, realized point values, coefficients, local deviation rules, financing levels, and case mix differ across regions, so the findings cannot be generalized to all Anhui hospitals, other TCM hospitals, or general hospitals. Second, the post-implementation design lacked a pre-DIP baseline and a contemporaneous control, precluding causal attribution and determination of whether the pattern was TCM-specific. Third, the observed readmission indicator captured only returns to the same hospital and could not distinguish planned from unplanned events; mortality, complications, item-level TCM services, test volumes, prices, indications, and special-case negotiation uptake and outcomes were unavailable. Fourth, annual changes in source-field definitions may have introduced residual information bias. Fifth, the exact year-specific local formula and cut-offs underlying administrative deviation labels were unavailable and could not be independently validated. *Post hoc* expenditure-per-point cut-points were used only for data-consistency assessment. Finally, clustering and department ICCs address correlated observations but do not eliminate unmeasured confounding or convert descriptive associations into causal effects. Future multicenter controlled studies should integrate verified local algorithms, longitudinal cross-provider outcomes, item-level TCM services, service volumes and prices, and broader clinical-quality measures.

## Conclusion

At this single tertiary TCM hospital, increases in DIP-weighted case mix from 2022 to 2024 coincided with shorter average length of stay and lower mean inpatient cost but less favorable administrative settlement outcomes. The increase in examination and laboratory cost share was broadly distributed across departments, and the within-department component accounted for most of the hospital-level change; the available data cannot identify changes in volume, prices, clinical need, technology use, or strategic behavior as its cause. Patient- and department-clustered analyses supported the principal year, length-of-stay, disease-point, and payment-pathway associations, while showing that several small cost-share associations were less robust. The findings support targeted, auditable review with quality safeguards and TCM-specific measurement. Causal and generalizable conclusions require multicenter controlled studies with verified local rules and broader clinical outcomes.

## Data Availability

The de-identified data that support the findings of this study are not publicly available because they are owned by the Seventh Affiliated Hospital of Anhui University of Chinese Medicine and are subject to institutional data-governance and patient-confidentiality requirements. The de-identified data may be made available from the corresponding author (Juanke1220@163.com) upon reasonable request and subject to approval by the hospital and the relevant ethics or data-governance bodies. Aggregate supporting results are provided in the Supplementary materials.
