# Supplementary material for: Rising DIP-weighted case mix, restructuring of cost composition, and expanding settlement deficits under diagnosis-intervention packet payment: evidence from case-level data in a tertiary grade a traditional Chinese medicine hospital in Anhui Province, China, 2022–2024
Source: Front Public Health. 2026 Sep 8;14:1868226. doi: 10.3389/fpubh.2026.1868226 (PMC13593479; doi:10.3389/fpubh.2026.1868226)
Supplement: Supplementary file 1 [file Data_Sheet_1.PDF]

Table S1. Annual source-system fields, analytic coding, and post hoc concordance assessment for disease-specific payment-pathway status and DIP deviation categories, 2022 – 2024.

Panel A. Disease-specific payment-pathway status recorded in the annual source systems.

Year	Source-system field	Original source-system values	Managed under a disease-specific payment pathway, n (%)	Not managed under a disease-specific payment pathway, n (%)
2022	Single-disease	Yes: 13,868; same disease, same coverage: 356; No: 37,252	14,224 (27.6)	37,252 (72.4)
2023	Whether single-disease	Yes: 11,121; No: 49,666	11,121 (18.3)	49,666 (81.7)
2024	Whether single-disease	Yes: 9,421; No: 54,569	9,421 (14.7)	54,569 (85.3)

Panel B. System-recorded deviation categories and post hoc concordance assessment.

Year	Source-system field	Total cases, N	Normal group, n (%)	High-multiple group, n (%)	Low-multiple group, n (%)	Cases with valid disease point values, N (%)	Concordant with post hoc operational rule, n/N (%)	Discordant, n/N (%)
2022	Deviation flag	51,476	41,527 (80.7)	5,352 (10.4)	4,597 (8.9)	50,476 (98.1)	47,583/50,476 (94.3)	2,893/50,476 (5.7)
2023	Deviation	60,787	50,114 (82.4)	2,950 (4.9)	7,723 (12.7)	60,787 (100.0)	59,674/60,787 (98.2)	1,113/60,787 (1.8)
2024	Cost category	63,990	54,462 (85.1)	1,688 (2.6)	7,840 (12.3)	63,990 (100.0)	61,932/63,990 (96.8)	2,058/63,990 (3.2)

Panel C. Classification frameworks and their role in the study.

Framework	Formula and denominator	Low-multiple category	Normal category	High-multiple category	Role in this study
National technical specification	$r_i = C_i/Sg,t$; Sg,t is the payment standard for the assigned DIP disease combination in the relevant pooling region and settlement year	$r_i < 0.50$	$0.50 \leq r_i \leq 2.00$	$r_i > 2.00$	Policy benchmark only; not used to reconstruct the study outcome
Annual local settlement-system classification, 2022–2024	Administrative category recorded in the annual hospital export; the exact local formula and annual cut-offs were not contained in the case-level dataset	System label: low-multiple	System label: normal	System label: high-multiple	Primary classification used in all descriptive and regression analyses
Post hoc data-consistency rule	$q_i = C_i/P_i$; P_i is the assigned disease point value; unit: CNY/point	$q_i < 33.3$	$33.3 \leq q_i \leq 133.2$	$q_i > 133.2$	Concordance audit only; never used to reassign system-recorded categories

Notes: DIP, Diagnosis-Intervention Packet; TCM, traditional Chinese medicine; CNY, Chinese yuan. C_i is the total inpatient medical expenditure for case i; P_i is the assigned DIP disease point value; and Sg,t is the payment standard for the assigned DIP disease combination in the relevant pooling region and settlement year.

(1) The source-system categories, rather than reconstructed categories, were used in all substantive analyses. The post hoc rule was used solely to assess reconstruction agreement and should not be interpreted as an independently verified local policy parameter.

(2) No observation had a q_i value exactly equal to 33.3 or 133.2 CNY/point; therefore, the source-system convention at the exact boundaries could not be inferred from the exported data.

(3) In 2022, disease point value was missing for 1,000 cases (527 low-multiple, 470 normal, and 3 high-multiple). These cases were retained in the source-system category counts but excluded from the post hoc concordance assessment.

(4) Discordant classifications comprised 2,822 core-disease, 62 comprehensive-disease, and 9 special-case-negotiation cases in 2022; 650 A+B-disease, 457 core-disease, and 6 severe-rehabilitation cases in 2023; and 1,166 A+B-disease, 459 core-disease, 432 TCM-advantaged-disease, and 1 severe-rehabilitation case in 2024.

(5) The national technical specification identifies extremely low-cost cases as those below 50% of the assigned disease-combination payment standard and extremely high-cost cases as those exceeding twice that standard. The national operating procedures permit the pooling region to determine the applicable deviation proportion. These national thresholds are contextual benchmarks, not verified Fuyang-specific thresholds.

Table S2. Annual examination and laboratory cost measures, 2022–2024.

Measure	2022	2023	2024	2022–2024 change
Admissions, n	51,476	60,787	63,990	+24.3%
Examination/laboratory cost, median (P25, P75), CNY	1,214.78 (730.77, 1,883.79)	1,150.60 (649.40, 1,900.70)	1,221.90 (670.10, 1,973.65)	+7.13 CNY (+0.59%)
Examination/laboratory cost, mean, CNY	1,423.37	1,418.11	1,545.76	+8.60%
Total medical expenditure, median (P25, P75), CNY	5,221.94 (3,484.03, 7,979.58)	4,738.59 (3,125.29, 7,278.38)	4,658.85 (3,126.80, 7,202.48)	−563.10 CNY (−10.78%)
Total medical expenditure, mean, CNY	7,376.70	6,767.01	6,823.18	−7.50%

Measure	2022	2023	2024	2022–2024 change
Case-level share, median (P25, P75), %	22.0 (14.0, 34.3)	22.7 (14.4, 35.7)	24.5 (15.3, 38.8)	+2.53 pp
Hospital aggregate share, %	19.30	20.96	22.65	+3.36 pp

Note: Case-level shares are summarized across admissions. Hospital aggregate shares are calculated from annual component and total-cost sums. CNY, Chinese yuan; P25, 25th percentile; P75, 75th percentile; pp, percentage points.

Table S3. Department-level aggregate examination and laboratory cost shares, 2022 versus 2024.

Stable department	2022 n	2022 share, %	2024 n	2024 share, %	Change, pp
Cardiac Intensive Care	205	10.51	342	22.25	+11.73
Cardiology II	1,627	19.96	1,981	29.76	+9.80
Gynecology	873	21.01	2,539	30.63	+9.63
Intensive Care	262	10.12	238	19.12	+9.00
Neurology II	2,418	24.22	3,134	30.80	+6.58
Cardiology I	2,383	24.43	2,749	30.34	+5.90
Pulmonology	2,031	19.74	2,626	25.28	+5.54
Nephrology	1,529	20.79	1,799	25.90	+5.11
Orthopedics II	791	14.94	914	19.47	+4.54
Surgery III	1,550	17.34	1,190	21.73	+4.39
Surgery I	2,408	17.41	2,324	21.57	+4.16
Gastroenterology	1,405	24.39	1,540	28.35	+3.96
Radiotherapy	960	11.68	923	15.23	+3.55
Orthopedics V	549	19.18	785	22.70	+3.52
General & Geriatric Medicine	1,268	25.35	1,524	28.33	+2.98
Geriatrics	662	18.99	1,138	21.93	+2.94
Orthopedics VI	470	16.94	758	19.85	+2.91
Ophthalmology	625	14.16	827	16.87	+2.72
Oncology	3,215	22.92	3,557	25.09	+2.16
Obstetrics & Gynecology	1,352	24.58	825	26.51	+1.93
Orthopedics VII	436	14.36	777	16.26	+1.90
Orthopedics IV	511	15.01	656	16.87	+1.85
Orthopedics III	335	17.48	566	19.19	+1.71
Surgery II	1,400	21.27	1,723	22.93	+1.66
Rehabilitation	808	6.65	1,231	8.32	+1.66
Neurology III	1,341	28.89	1,358	30.54	+1.64
Orthopedics I	650	16.13	885	17.60	+1.47
Otolaryngology	2,540	16.57	2,410	17.87	+1.30
Hepatology	934	14.76	1,013	15.86	+1.10
Anorectal Surgery	1,672	17.45	2,418	18.03	+0.58
Pediatrics I	2,469	18.26	3,553	18.61	+0.35

Stable department	2022 n	2022 share, %	2024 n	2024 share, %	Change, pp
Emergency Medicine	189	24.06	745	24.34	+0.28
Tuina I	628	9.34	747	9.55	+0.21
Rheumatology	770	34.06	983	34.24	+0.19
Pediatrics II	1,555	22.70	1,998	22.56	-0.13
Tuina II	696	10.11	881	9.67	-0.44
Neurology I	2,623	31.35	3,102	29.37	-1.98
Neonatal Intensive Care	995	25.57	809	23.14	-2.42
Acupuncture	890	11.58	1,013	8.82	-2.76
Endocrinology	1,332	28.75	1,657	25.74	-3.01
Dermatology	1,365	14.19	1,701	10.18	-4.01

Note: Stable departments had at least 100 admissions in each year after prespecified department-name harmonization. Thirty-four of 41 stable departments increased and seven decreased. pp, percentage points.

Table S4. Exact-code-matched DIP disease-group sensitivity analysis.

DIP group code	2022 n	2023 n	2024 n	2022 share, %	2024 share, %	Change, pp
I67.8:n(y)	2,148	3,305	2,377	41.04	42.98	+1.95
J15.9:n(y)	648	2,900	2,764	20.47	20.57	+0.10
E11.7:n(y)	1,137	1,521	1,155	29.09	25.20	-3.88
I63.5:n(y)	1,121	1,319	1,291	27.92	30.41	+2.49
Z51.1:99.2503	688	1,030	1,599	22.59	25.79	+3.20
J44.1:n(y)	1,083	1,081	1,004	17.70	24.63	+6.93
M51.1:n(y)	861	1,344	140	9.64	18.06	+8.42
I50.1:n(y)	566	898	813	23.81	32.85	+9.04
Z51.1:n(y)	1,378	617	251	26.73	30.73	+4.00
I25.1:n(y)	648	722	579	34.78	43.89	+9.11
Z51.8:n(y)	975	484	372	24.58	33.92	+9.34
I50.9:n(y)	633	505	538	26.46	34.94	+8.48
J98.4:n(y)	208	409	1,028	20.13	23.86	+3.73
J18.9:n(y)	900	360	166	16.70	23.14	+6.44
Z51.8:99.2800x006	266	541	608	12.36	13.69	+1.33
K64.8:17.98330	477	578	331	12.29	10.02	-2.27
B99.x:n(y)	130	414	816	36.08	28.04	-8.04
K29.1:n(y)	445	524	336	26.25	26.19	-0.05
I63.3:n(y)	356	472	443	29.52	32.14	+2.62
P23.9:n(y)	430	381	444	26.54	24.74	-1.80
Z51.5:n(y)	397	556	248	28.65	34.99	+6.34
N18.5:n(y)	446	405	182	19.68	24.56	+4.89
I63.9:n(y)	689	194	146	26.41	28.87	+2.46

DIP group code	2022 n	2023 n	2024 n	2022 share, %	2024 share, %	Change, pp
G45.9:n(y)	434	356	233	43.01	47.61	+4.60
K56.7:n(y)	275	299	435	28.08	27.55	-0.53
I24.9:n(y)	414	324	264	34.21	38.93	+4.72
J20.9:n(y)	292	516	181	17.88	20.63	+2.74
J98.7:n(y)	184	470	192	26.32	28.29	+1.97
K80.1:51.2300	324	301	196	18.60	24.85	+6.26
A49.3:n(y)	110	423	243	18.25	21.41	+3.16
N04.9:n(y)	289	256	213	26.18	31.42	+5.24
G51.8:n(y)	297	346	113	10.63	19.28	+8.64
I20.0:n(y)	120	246	336	39.70	42.37	+2.67
H81.3:n(y)	201	218	251	40.60	46.55	+5.95
J21.9:n(y)	199	260	199	15.61	17.34	+1.73
I25.1:88.5500	156	197	250	19.44	54.77	+35.33
S72.1:79.1500x006	156	184	243	16.77	21.58	+4.81
K35.8:47.0100	258	129	173	17.16	22.47	+5.32
G20.x:n(y)	171	164	215	28.89	34.33	+5.44
L50.8:n(y)	124	154	231	24.95	15.95	-9.00
H91.2:n(y)	164	183	160	16.52	14.88	-1.64
S72.0:81.5201	155	142	180	14.62	16.04	+1.42
M17.9:81.5400	154	188	135	12.02	15.40	+3.39
Z51.0:92.2400x003	188	158	125	6.57	9.14	+2.57
K92.2:n(y)	190	115	137	24.93	29.99	+5.06
K74.6:n(y)	133	111	179	17.86	17.36	-0.51
H81.1:n(y)	131	154	137	43.32	50.37	+7.05
K85.9:n(y)	134	157	103	22.82	26.58	+3.75

Note: Only exact normalized codes observed in all three years with at least 100 admissions per year are shown (48 codes). These codes covered 42.5%, 43.8%, and 35.6% of admissions in 2022, 2023, and 2024. Because no crosswalk for annual catalogue revisions was available, this is a sensitivity analysis rather than the primary subgroup analysis. DIP, Diagnosis-Intervention Packet; pp, percentage points.

Table S5. Sensitivity analyses for the 2022 − 2024 aggregate-share change.

Analysis	2022 n	2023 n	2024 n	Observed change, pp	Within, pp	Between, pp
Full sample	51,476	60,787	63,990	+3.359	+3.435	-0.076
Exclude top 1% total cost within year	50,961	60,179	63,350	+3.309	+3.658	-0.350
Exclude top 1% length of stay within year	50,966	60,224	63,409	+3.235	+3.317	-0.082
Exclude both year-specific upper 1% tails	50,651	59,819	63,008	+3.264	+3.599	-0.336

Note: Upper-tail exclusions were applied separately within each year. The direction and magnitude of the aggregate increase were retained. pp, percentage points.

Table S6. Prespecified department-name harmonization rules.

Source labels	Harmonized analytical label
General Medicine and Geriatric Diseases	General & Geriatric Medicine
Gynecology I; Gynecology II	Gynecology
Geriatrics I; Geriatrics II	Geriatrics
Rehabilitation I; Rehabilitation II; Rehabilitation III	Rehabilitation
Anorectal Surgery II	Anorectal Surgery
Pulmonary and Critical Care Medicine II; III; IV	Pulmonology

Note: English translations are shown for readability; the exact UTF-8 Chinese source-label mapping and its reproducible implementation are provided in Supplementary Code S1. All other department labels were retained after trimming leading and trailing whitespace. Department-name harmonization was prespecified and applied consistently across the cost-composition, department, clustering, and sensitivity analyses.

Table S7. Direction-specific DIP deviation classifications in the 20 highest-volume departments, 2022–2024.

Rank	Department	Total, n	Normal, n (%)	High-multiple, n (%)	Low-multiple, n (%)	Any deviation, n (%)
1	Pediatrics I	10,143	8,402 (82.8)	134 (1.3)	1,607 (15.8)	1,741 (17.2)
2	Oncology	9,959	7,034 (70.6)	667 (6.7)	2,258 (22.7)	2,925 (29.4)
3	Neurology I	8,718	7,579 (86.9)	506 (5.8)	633 (7.3)	1,139 (13.1)
4	Neurology II	8,655	7,661 (88.5)	373 (4.3)	621 (7.2)	994 (11.5)
5	Cardiology I	7,713	6,853 (88.8)	204 (2.6)	656 (8.5)	860 (11.2)
6	Otolaryngology	7,695	6,755 (87.8)	201 (2.6)	739 (9.6)	940 (12.2)
7	Surgery I	7,691	6,431 (83.6)	366 (4.8)	894 (11.6)	1,260 (16.4)
8	Pulmonology	7,307	6,192 (84.7)	502 (6.9)	613 (8.4)	1,115 (15.3)
9	Anorectal Surgery	6,054	3,854 (63.7)	1,352 (22.3)	848 (14.0)	2,200 (36.3)
10	Pediatrics II	5,905	5,163 (87.4)	72 (1.2)	670 (11.3)	742 (12.6)
11	Cardiology II	5,571	4,851 (87.1)	109 (2.0)	611 (11.0)	720 (12.9)
12	Nephrology	4,967	3,425 (69.0)	225 (4.5)	1,317 (26.5)	1,542 (31.0)
13	Endocrinology	4,642	4,257 (91.7)	107 (2.3)	278 (6.0)	385 (8.3)

Rank	Department	Total, n	Normal, n (%)	High-multiple, n (%)	Low-multiple, n (%)	Any deviation, n (%)
14	Surgery II	4,606	3,973 (86.3)	131 (2.8)	502 (10.9)	633 (13.7)
15	Gynecology	4,604	4,194 (91.1)	184 (4.0)	226 (4.9)	410 (8.9)
16	Dermatology	4,593	3,898 (84.9)	406 (8.8)	289 (6.3)	695 (15.1)
17	Spleen and Stomach Diseases	4,576	3,930 (85.9)	228 (5.0)	418 (9.1)	646 (14.1)
18	Surgery III	4,399	2,777 (63.1)	429 (9.8)	1,193 (27.1)	1,622 (36.9)
19	General & Geriatric Medicine	4,378	3,698 (84.5)	401 (9.2)	279 (6.4)	680 (15.5)
20	Neurology III	4,051	3,624 (89.5)	219 (5.4)	208 (5.1)	427 (10.5)

Note: Departments were ranked by cumulative admission volume after applying the same prespecified harmonization used in Table 3 and Figure 3. Categories are administrative source-system labels. Direction-specific proportions are descriptive and do not identify coding, clinical, efficiency, or gaming mechanisms. DIP, Diagnosis-Intervention Packet.

Table S8. Repeated-admission structure and department-level clustering.

Measure	Result	Definition/interpretation
Admissions	176,253	Analytical unit
Unique patients	118,520	Patient clusters in the full sample
Patients with multiple admissions	27,912 (23.6%)	At least two admissions during the study data window
Admissions contributed by repeat patients	85,645 (48.6%)	Admissions belonging to patients with at least two records
Patients observed in more than one settlement year	17,120	Longitudinal dependence across annual extracts
Complete-case admissions / patient clusters	175,253 / 117,905	Regression sample after excluding missing disease point value
Harmonized departments	51	Department clusters after Table S6 mapping
Department ICC: settlement deficit	0.080	Intercept-only logistic random-intercept model
Department ICC: any-deviation status	0.107	Intercept-only logistic random-intercept model

Note: The main models use two-way patient- and department-clustered sandwich covariance estimates. Department ICCs were estimated from intercept-only logistic random-intercept models using adaptive Gauss–Hermite quadrature. ICC = $\sigma^2/(\sigma^2 + \pi^2/3)$. ICC, intraclass correlation coefficient.

Table S9. Restricted cubic spline tests for nonlinearity of age and length of stay.

Outcome	Variable	Knots	Two-way cluster-robust Wald χ^2	df	P for nonlinearity
Settlement deficit	Age	2, 50, 67, 83	5.48	2	0.065
Settlement deficit	Length of stay	2, 6, 9, 21	18.68	2	<0.001
Any-deviation status	Age	2, 50, 67, 83	1.52	2	0.467
Any-deviation status	Length of stay	2, 6, 9, 21	68.86	2	<0.001

Note: Knots were placed at the 5th, 35th, 65th, and 95th percentiles. Wald tests evaluated the joint significance of the two nonlinear spline terms using the same two-way patient- and department-clustered covariance as the main models. The linear odds ratios in Tables 4 and 5 are therefore compact average-slope summaries; where nonlinearity was detected, they should not be assumed constant over the observed range. df, degrees of freedom.

Table S10. Multivariable regression sensitivity analyses excluding extreme total-cost and length-of-stay observations.

Panel A. Case-level DIP settlement deficit.

Analysis	n	2023 vs 2022	2024 vs 2022	Pathway yes vs no	LOS per day	Points per 10	Western share per 10 pp	Exam/lab share per 10 pp	Material share per 10 pp	Therapeutic share per 10 pp
Full sample	175,253	1.206 (1.033–1.408)	1.424 (1.206–1.682)	0.366 (0.276–0.485)	1.123 (1.080–1.168)	0.946 (0.930–0.962)	1.035 (0.923–1.160)	0.879 (0.785–0.984)	1.071 (0.920–1.247)	0.860 (0.793–0.933)
Exclude top 1% total cost	173,500	1.217 (1.041–1.423)	1.459 (1.231–1.730)	0.359 (0.270–0.478)	1.136 (1.088–1.186)	0.930 (0.912–0.948)	1.050 (0.934–1.181)	0.886 (0.790–0.995)	1.118 (0.948–1.320)	0.866 (0.796–0.942)
Exclude top 1% length of stay	173,631	1.212 (1.037–1.416)	1.438 (1.218–1.698)	0.362 (0.272–0.482)	1.135 (1.089–1.182)	0.944 (0.926–0.962)	1.039 (0.925–1.168)	0.887 (0.789–0.997)	1.079 (0.924–1.262)	0.863 (0.793–0.938)
Exclude both upper tails	172,506	1.220 (1.042–1.427)	1.466 (1.238–1.735)	0.357 (0.268–0.476)	1.143 (1.095–1.193)	0.930 (0.911–0.949)	1.050 (0.933–1.182)	0.891 (0.793–1.002)	1.117 (0.945–1.320)	0.867 (0.796–0.945)

Panel B. Any-deviation status.

Analysis	n	2023 vs 2022	2024 vs 2022	Pathway yes vs no	LOS per day	Points per 10	Western share per 10 pp	Exam/lab share per 10 pp	Material share per 10 pp	Therapeutic share per 10 pp
Full sample	175,253	0.855 (0.752–0.972)	0.659 (0.540–0.803)	0.384 (0.199–0.739)	0.986 (0.959–1.015)	1.014 (1.005–1.022)	1.188 (1.030–1.370)	1.134 (0.990–1.299)	0.744 (0.624–0.888)	1.043 (0.950–1.144)
Exclude top 1% total cost	173,500	0.828 (0.724–0.948)	0.638 (0.520–0.782)	0.385 (0.199–0.748)	0.978 (0.946–1.011)	1.028 (1.016–1.039)	1.173 (1.019–1.349)	1.131 (0.987–1.297)	0.628 (0.541–0.728)	1.054 (0.960–1.157)
Exclude top 1% length of stay	173,631	0.855 (0.754–0.969)	0.659 (0.540–0.804)	0.386 (0.201–0.742)	0.973 (0.937–1.010)	1.019 (1.010–1.028)	1.180 (1.023–1.362)	1.124 (0.976–1.294)	0.724 (0.605–0.867)	1.051 (0.957–1.155)
Exclude both upper tails	172,506	0.830 (0.727–0.949)	0.640 (0.522–0.785)	0.388 (0.200–0.753)	0.967 (0.930–1.006)	1.032 (1.020–1.045)	1.169 (1.016–1.345)	1.124 (0.977–1.293)	0.615 (0.531–0.712)	1.060 (0.966–1.164)

Note: Values are odds ratios (95% confidence intervals) from the same covariate set as the main models, with two-way patient- and department-clustered standard errors. Upper-tail exclusions were applied separately within settlement year. The principal year, disease-specific pathway, and disease-point directions were retained; some cost-share confidence intervals crossed the null. pp, percentage points; LOS, length of stay.

Table S11. Observed 30-day all-cause readmission to the same hospital by discharge year.

Discharge year	Eligible index discharges, n	Readmissions within 30 days, n	Rate, %
2022	51,474	5,756	11.18
2023	60,785	6,622	10.89
2024	59,026	6,809	11.54

Note: $\chi^2 = 12.42$, df = 2, P = 0.002. The maximum absolute annual difference was 0.64 percentage points. This measure includes only admissions to the study hospital and cannot distinguish planned from unplanned readmissions; it should not be treated as a comprehensive quality endpoint. df, degrees of freedom.

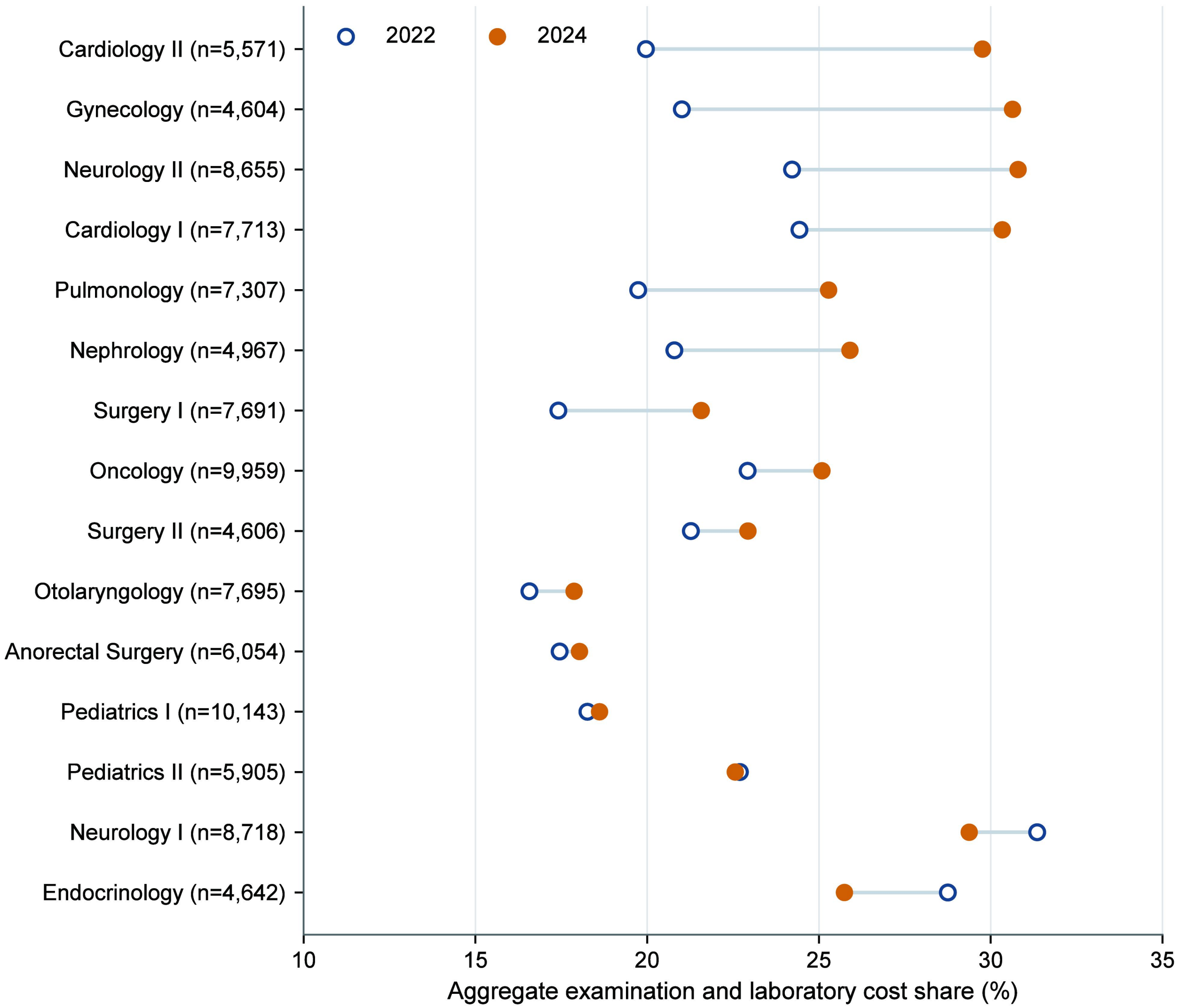

Figure S1. Examination and laboratory cost share in the 15 highest-volume stable departments, 2022 and 2024.

Note: The share for each department-year is Σ examination and laboratory cost/Σ total medical expenditure. Departments were selected by pooled 2022–2024 admission volume before ranking any outcome. Open blue circles indicate 2022; filled orange circles indicate 2024.

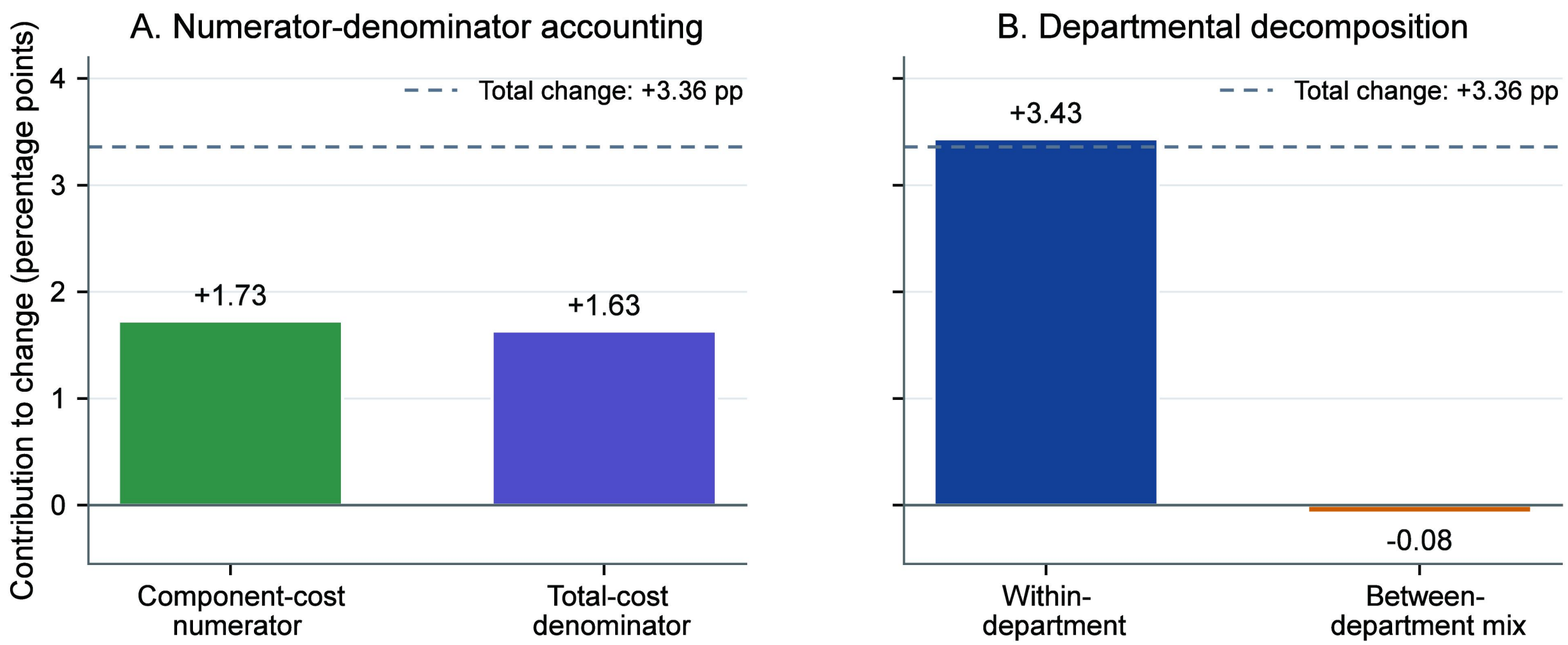

Figure S2. Accounting decompositions of the 2022–2024 change in the hospital aggregate examination and laboratory cost share.

Note: Panel A uses an exact symmetric decomposition of the aggregate ratio into the change in mean component cost and the change in mean total expenditure. Panel B uses the exact within-/between-department decomposition. Contributions are accounting identities, not causal effects. pp, percentage points.
